# International Collaborative Study on Human Papillomavirus Analytical Thresholds for Sensitivity and Specificity in Cervical Screening

**DOI:** 10.1002/jmv.70936

**Published:** 2026-04-25

**Authors:** Emel Yilmaz, Gerald Laurence Murray, Prisha Balgovind, Suzanne Marie Garland, Rita Pereira, Davy Vanden Broeck, Nina Redzic, Jean‐Luc Prétet, Quentin Lepiller, Steffi Silling, Clementina Cocuzza, Marianna Martinelli, Allan Campbell, Conor Brown, Kate Cuschieri, Linzi Connor, Anja Oštrbenk, Mario Poljak, Murat Gultekin, Yalın Kılıç, Klara Miriam Elfström, Laila Sara Arroyo Mühr, Joakim Dillner

**Affiliations:** ^1^ International HPV Reference Center, Center for Cervical Cancer Elimination Stockholm Sweden; ^2^ Department of Clinical Science, Intervention and Technology (CLINTEC), Center for Cervical Cancer Elimination Karolinska Institutet Stockholm Sweden; ^3^ Department of Obstetrics, Gynaecology and Newborn Health The University of Melbourne Melbourne VIC Australia; ^4^ Murdoch Children's Research Institute Melbourne VIC Australia; ^5^ Women's Centre for Infectious Diseases The Royal Women's Hospital Melbourne VIC Australia; ^6^ Laboratory of Molecular Diagnostics, AML Sonic Healthcare Benelux Antwerp Belgium; ^7^ National Reference Centre for HPV Brussels Belgium; ^8^ Université Marie et Louis Pasteur, CNRS Chrono‐environnement (UMR 6249) Besançon France; ^9^ Centre National de Référence Papillomavirus CHU Besançon France; ^10^ Institute of Virology, National Reference Centre for Papilloma‐ and Polyomaviruses, University of Cologne Faculty of Medicine and University Hospital Cologne Cologne Germany; ^11^ School of Medicine and Surgery University of Milano‐Bicocca Milan Italy; ^12^ Scottish Human Papillomavirus Reference Laboratory, Royal Infirmary of Edinburgh Edinburgh UK; ^13^ Institute of Microbiology and Immunology, Faculty of Medicine University of Ljubljana Ljubljana Slovenia; ^14^ Hacettepe University Faculty of Medicine Department of Gynaecological Oncology Ankara Turkey; ^15^ Department of Biomedical Engineering Acıbadem Mehmet Ali Aydınlar University Istanbul Turkey

**Keywords:** analytical thresholds, cervical screening, HPV, HPV virus amount, human papillomavirus, virus amounts

## Abstract

Human papillomavirus (HPV) assays vary regarding the minimum amount of virus they detect. We investigated analytical thresholds of HPV detection and cervical screening sensitivity and specificity. One hundred cervical intraepithelial neoplasia grade 2 or worse (CIN2+) cases and 200 matched population‐based controls were obtained at the Swedish National HPV Reference Laboratory and analyzed by 10 laboratories across 10 countries. Cumulative sensitivity (weighted according to the global HPV type distribution in invasive cervical cancer (ICC)) and specificity were estimated at varying analytical detection thresholds. Consensus results found HPV in 99/100 CIN2+ cases and 52/200 controls. HPV16 prevalence declined in HPV‐vaccinated birth cohorts, among both cases and controls. Line plots of 1‐specificity and ICC‐weighted sensitivity found optimal analytical detection thresholds as 3 International Units (IU)/µl for HPV16/18, 25 IU/µl for HPV31/33/35/45/52/58 and 100 genome equivalents (GE)/µl for HPV 39/51/56/59 resulting in 92.00% cumulative specificity and 90.08% ICC‐weighted sensitivity. Thresholds defined using virus amounts per 10^4^ human cells gave similar results. Comparator assay testing using manufacturer‐defined thresholds achieved high ICC‐weighted sensitivity (96.61%) but low specificity (82.50%). This international collaborative study has identified HPV analytical detection thresholds optimizing the sensitivity and specificity of cervical screening.

**Abbreviations**
CCCE: Center for Cervical Cancer Elimination
CCR5: C‐C motif chemokine receptor 5
CIN2+: Cervical intraepithelial neoplasia grade 2 or worse, including adenocarcinoma in situ and invasive cervical cancer
Ct: Cycle thresholds
GE: Genome Equivalent
HPV: Human papillomaviruses
HSIL: High‐grade Squamous Intraepithelial Lesion
IARC: International Agency for Research on Cancer
ICC: Invasive cervical cancer
IS: International Standard
IU: International Unit
LBC: Liquid‐based Cytology
LSIL: Low‐grade Squamous Intraepithelial Lesion
NRL: National Reference Laboratory
PAF: Population Attributable Fraction
TPP: Target Product Profile
WHO: World Health Organization

## 1 Introduction

Cervical screening based on human papillomavirus (HPV) testing is more sensitive and objective than cytology in preventing invasive cervical cancer (ICC) [1]. The World Health Organization (WHO) has named high‐performance HPV‐based screening as a pillar of the global strategy to eliminate cervical cancer [2].

High amounts of HPV DNA are associated with infection persistence, with low amounts linked to transient infections [3, 4]. High HPV amounts may also be associated with progression to cervical intraepithelial neoplasia grade 2 or worse (CIN2+) [5, 6, 7, 8, 9]. This has been consistently observed for HPV16 [10, 11, 12]. A prospective cohort study with ICC as endpoint, reported optimal sensitivity and specificity when HPV16/18 were detected at low quantities, but higher analytical thresholds were used for HPV 31/33/45/52 [13]. Although early ICC detection is one purpose of screening, the main purpose is identifying and treating CIN2+ lesions.

More than 260 different HPV tests are commercially available (in addition to numerous in‐house methods) [14]. However, performances vary, both by assay and by the laboratory performing the tests [15, 16, 17, 18, 19, 20, 21, 22, 23, 24]. Criteria for validation of HPV assays have focused on clinical sensitivity and specificity for detection of CIN2+, without consideration of the amount of virus that should be detectable. We launched an international collaborative study involving expert laboratories from multiple countries, including national HPV reference laboratories (NRL). An international consensus on required analytical thresholds for HPV assays would be important for quality assurance and continued development and validation of HPV assays.

## 2 Materials and Methods

The Swedish cervical screening program mandates HPV‐based screening for all women ages between 23–70 years and uses reflex cytology after HPV‐positivity. Women with cytological abnormalities are referred for cervical biopsy and histopathology. For this study, cases and controls were identified by the Center for Cervical Cancer Elimination (CCCE), the central laboratory for the regional population‐based screening program. CCCE registers the HPV tests, cytology, and histopathology results. Cases were women with CIN2+ in histopathology with a liquid‐based cytology (LBC) sample registered at the same date or at most 3 months before the histopathology sampling for CIN2+ diagnosis. Controls were women participating in the population‐based screening program who had LBC samples taken for primary screening but no histopathology or CIN2+ in histopathology during the same follow‐up period as the cases. For each case, two controls were selected, matched by age (±5years). 100 consecutive eligible cases and 200 age‐matched controls were identified between 4th April 2022 and 27th March 2023. The mean ages were 34.33 years (cases) and 35.40 years (controls).

We prepared a dilution series of HPV DNA (whole genome HPV plasmids) for each of the 12 HPV types that are classified as oncogenic by the International Agency for Research on Cancer (IARC): HPV16/18/31/33/35/39/45/51/52/56/58/59 [25]. For HPV16/18/31/33/45/52/58 we used International Standards (IS) of HPV DNA and report concentrations in International Units (IU)/µl. For HPV35/39/51/56/59, dilution series used defined amounts of genome equivalents (GE)/µl of HPV DNA. The ten‐fold dilutions ranged from 10^5^ to 10^0^ IU or GE per µl, yielding a total of 72 standards for calibration. HPV DNA was diluted in TE buffer (10 mM TRIS‐HCL, 0.1 mM EDTA, pH 8.0) containing 10 ng/µl human DNA.

Study sample aliquots and standards were distributed to expert laboratories in 10 countries participating in the global HPV LabNet (Australia, Belgium, France, Germany, Italy, Scotland, Slovenia, Sweden, Türkiye and USA). Each aliquot was anonymized with a unique identifier and shipped blinded, without any clinical, pathological or virological information. The participating laboratories performed analyses using their extraction and HPV testing methods, as detailed in Supporting Information 1.

From 10 countries, 13 datasets were obtained, as some laboratories used more than one assay. Germany and Sweden performed virus quantification only on HPV‐positive samples. For consensus on the presence of HPV, we required ≥ 70% agreement regarding the detected HPV type. If multiple HPV types were detected in the same sample, positivity was assigned following hierarchy of cancer risk among class I carcinogen viruses [25]: HPV16 > 18 > 45 > 33 > 58 > 31 > 52 > 35 > 59 > 39 > 51 > 56 > Other HPV types. Several labs also reported positivity for viruses not established as oncogenic: HPV6/40/42/44/54/61/66/68/70/82 which in this study are termed as “Other HPV”. Cumulative sensitivity and specificity for detecting a case were first calculated for each HPV type with reported interpretation as positive even if they corresponded to extremely low virus amounts.

Cycle threshold (Ct) values from serially diluted standards, were utilized to generate standard curves for HPV types 16/18/31/33/35/39/45/51/52/56/58/59. Two laboratories did not detect standards at the lowest concentrations, therefore only data from five ten‐fold dilutions (10^5^–10^1^ IU or GE per µl) were included from their datasets. Some laboratories reported additional HPV types that should not be present in the standards. A previously reported contamination with low amounts of HPV35 in the HPV31 standard was confirmed [24], whereas all other false positives were limited to the reporting laboratory.

Seven laboratories provided Ct values for both calibration and clinical samples, enabling quantification of viral amounts using standard curves. Theoretical concentrations of the standards were log‐transformed, and linear regression was performed with Ct values as the dependent variable and log10 concentrations as the independent variable, yielding slope, intercept, and R‐squared values. White's test was used to assess heteroscedasticity. HPV type‐specific virus amounts in each HPV‐positive sample were calculated as 10$\frac{\left(\mathrm{Ct}-\mathrm{intercept}\right)}{\mathrm{slope}}$ [26, 27]. Virus amounts per µl of original sample volume were calculated based on the input sample volume in extraction and volume of the resulting extracted DNA material (Table SI). For each HPV type, median viral amounts were determined for datasets that were positive for the consensus virus by seven laboratories. As many CIN lesions are caused by HPV types with limited contribution to ICC, sensitivities were weighted according to the importance of each virus in the etiology of ICC using global attributable fractions [25]. Cumulative sensitivity, cumulative specificity, and ICC‐weighted sensitivity were calculated.

**Figure 1:**
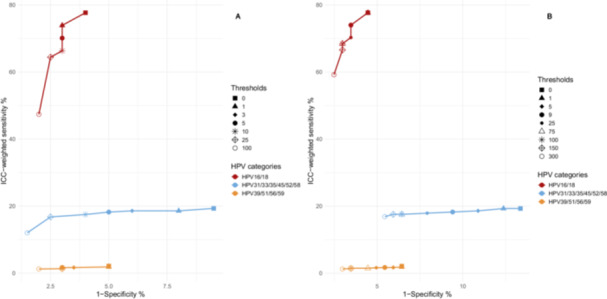
Line plots comparing ICC weighted sensitivity and 1‐specificity based on varying thresholds (A) per µl of sample volume for detection of three main categories of HPV types and (B) per 10^4^ cells for detection of three main categories of HPV types.

Quantitative assessment of human DNA used C‐C motif chemokine receptor five (CCR5) quantification was performed by the expert laboratory from Italy, providing both sample adequacy assessment and the number of cells/µl of original sample volume [28] (half of the quantity of CCR5 copies/µl). Virus amounts per 10^4^ cells were based on the input volume for DNA extraction and volume of the resulting extract. HPV amounts per 10^4^ cells were calculated by dividing the HPV amount per µl of original sample volume to cell count and thereafter multiplied by 10^4^. Line plots evaluated the relationship between 1‐specificity and ICC‐weighted sensitivity across varying analytical detection thresholds, by HPV amounts/µl of original sample volume and by HPV amounts per 10^4^ cells (Figure 1). All statistical analyses used STATA, version 17 and the figure was generated using R version 4.3.2.

## 3 Results

In the routine primary HPV screening (using BD Onclarity and Cobas 4800), 99/100 cases (99.00%) and 37/200 controls (18.50%) were reported as HPV‐positive. The Cobas 4800 assay reported results only in three channels (HPV16, HPV18 and non‐16/18 oncogenic HPV). Samples reported as positive for non‐16/18 oncogenic HPV were re‐analyzed using BD Onclarity for extended genotyping, of those two samples were HPV negative in BD. Reflex cytologies were abnormal for 94/100 cases. Among controls, there were eight abnormal and 31 normal cytologies. For 161 controls, there was no cytology (because of HPV negativity). The histopathologies among cases were 94 high‐grade squamous intraepithelial lesions (HSIL), two adenocarcinomas in situ (AIS), two AIS/HSIL and two squamous cell carcinomas (SCC). Among controls, there were two low‐grade squamous intraepithelial lesions (LSIL) and six normal histopathologies (remaining controls had no biopsy).

Consensus results from 13 datasets found HPV in 52/200 (26.00%) controls and 99/100 (99.00%) cases (all reported HPV types were considered, including detection of possibly/probably or not oncogenic HPV types), resulting in cumulative sensitivity and specificity of 99.00% and 74.00%, respectively (Table 1). Stratifying by birth cohorts with or without organized catch‐up vaccination with quadrivalent HPV vaccine (HPV6/11/16/18; population coverage ~55%) [29], vaccinated birth cohorts had 6/32 HPV16 positive cases (18.75%), compared with 28/68 cases (41.18%) among women born 1954–1962 (*p* = 0.041, Fischer's exact test among cases).

**Table 1:** Consensus on reference HPV type distribution by cases and controls determined from 13 datasets from 10 participating laboratories. Thresholds for virus amounts were not applied; the interpretation of the testing assays was approved (as it was done for BD Onclarity assay) and in case of raw data all signals were accepted as positive for HPV. In case of positivity for multiple HPV types, the most oncogenic HPV type is listed.

HPV type	Total	Control	Case	Cumulative sensitivity%	Cumulative Specificity (%)
16	40	6	34	34.00	97.00
18	9	2	7	41.00	96.00
45	6	2	4	45.00	95.00
33	12	3	9	54.00	93.50
58	13	7	6	60.00	90.00
31	20	5	15	75.00	87.50
52	10	2	8	83.00	86.50
35	6	1	5	88.00	86.00
59	3	1	2	90.00	85.50
39	6	3	3	93.00	84.00
51	3	2	1	94.00	83.00
56	6	3	3	97.00	81.50
Other HPV^a^	17	15	2	99.00	74.00
Negative/Not detectable/No consensus	149	148	1	100.00	0.00
Total	300	200	100		

a HPV 6, 40, 42, 44, (44, 61), 54, 61, 66, 68, (68, 70), 82.

Line plot inspection (Figure 1) showed that 3 IU/µl for HPV16 and 18 was required for adequate sensitivity while 25 IU/µl for HPV31/33/35/45/52/58 and 25 GE/µl for HPV35 gave an adequate sensitivity with limited adverse effect on specificity. For the lower oncogenicity HPV types (HPV39/51/56/59), their joint contribution to sensitivity was minimal, whereas detection at low thresholds gave a strong adverse effect on specificity (Figure 1). Using a threshold of 100 GE/µl for HPV39/51/56/59 improved specificity.

Table 2 compares the consensus HPV distribution in cases and controls with the HPV‐positives remaining after applying analytical thresholds. The number of HPV‐positives was reduced in particular among controls, increasing specificity. ICC‐weighted sensitivity of the major oncogenic HPV types 16/18/31/33/35/45/52/58 jointly was 88.72% (Table 2). When detection of HPV39/51/56/59 was included, ICC‐weighted sensitivity was 90.08% with a cumulative specificity of 92.00% (Table 2).

**Table 2:** Consensus HPV type distribution among seven participating laboratories by cases and controls with applied thresholds at 3 IU/μl of sample volume for HPV16 and 18; 25 IU/μl of sample volume for HPV31, 33, 35, 45, 52 and 58; 25 GE/μl of sample volume for HPV35; 100 GE/μl of sample volume for HPV39, 51, 56 and 59. In case of positivity for multiple HPV types, the most oncogenic HPV type is listed. ICC‐weighted sensitivity was calculated using population attributable fraction (PAF) adapted from IARC [25].

HPV type	Controls ‐ Reference	Controls – with thresholds	Cases ‐reference	Cases ‐ with thresholds	Controls remained positive after thresholds	Cases remained positive after thresholds	Sensitivity^b^	Cumulative sensitivity%^c^	Cumulative Specificity% c	PAF ICC%	Sensitivity with threshold%	Cumulative Sensitivity ICC weighted%
16	6	4	34	33	4	33	0.97	33.00	98.00	62.40	60.56	60.56
18	2	2	7	6	2	6	0.86	39.00	97.00	15.30	13.11	73.68
45	2	1	4	3	1	3	0.75	42.00	96.50	4.80	3.60	77.28
33	3	0	9	8	0	8	0.89	50.00	96.50	3.90	3.47	80.75
58	7	1	6	5	1	5	0.83	55.00	96.00	3.70	3.08	83.83
31	5	3	15	14	3	13	0.87	69.00	94.50	2.90	2.51	86.34
52	2	0	8	5	0	3	0.38	74.00	94.50	2.60	0.98	87.32
35	1	0	5	7	0	5	1.00	81.00	94.50	1.40	1.40	88.72
59	1	1	2	2	1	2	1.00	83.00	94.00	0.90	0.90	89.62
39	3	1	3	1	1	1	0.33	84.00	93.50	0.80	0.27	89.88
51	2	1	1	2	0	0	0.00	86.00	93.00	0.20	0.00	89.88
56	3	2	3	3	1	3	1.00	89.00	92.00	0.20	0.20	90.08
Other HPV^a^	15	—	2	—	—	—	—	89.00	92.00	—	—	—
Negative/Not detectable/No consensus	148	184	1	11	148	1		100.00	0.00			

a HPV 6, 40, 42, 44, (44 & 61), 54, 61, 66, 68, (68, 70), 82.

b Calculated as: cases remained positive after thresholds/cases ‐ reference.

c Based on HPV distribution among cases and controls after thresholds were applied.

Inspection of the line plot based on HPV amount per 10^4^ human cells revealed optimal sensitivity and specificity using the following thresholds: 9 IU/10^4^ human cells for HPV 16/18; 75 IU or GE/10^4^ human cells for HPV31/33/45/52/58/35 and 300 GE/10^4^ human cells for HPV39/51/56/59 (Figure 1). At these thresholds, cumulative specificity was 87.50% and ICC‐weighted sensitivity was 92.79% (Table 3).

**Table 3:** Data from expert laboratory in Italy showing HPV distribution among cases and controls with estimation of HPV virus amounts per 10^4^ cells. Applied thresholds were 9 IU/10^4^ cells for HPV16 and 18; 75 IU/10^4^ Cells for HPV31, 33, 35, 45, 52 and 58; 75 GE/10^4^ cells for HPV35; 300 GE/10^4^ Cells for HPV39, 51, 56 and 59. In case of positivity for multiple HPV types, the most oncogenic HPV type is listed. ICC‐weighted sensitivity was calculated using PAF adapted from IARC [25].

HPV type	Controls ‐ Reference	Controls – with thresholds	Cases ‐reference	Cases ‐ with thresholds	Controls remained positive after thresholds	Cases remained positive after thresholds	Sensitivity^a^	Cumulative sensitivity%^b^	Cumulative Specificity% b	PAF ICC%	Sensitivity with threshold%	Cumulative Sensitivity ICC weighted%
16	7	5	35	34	5	34	0.97	34.00	97.50	62.40	60.62	60.62
18	2	2	7	6	2	6	0.86	40.00	96.50	15.30	13.11	73.73
45	2	1	4	4	1	4	1.00	44.00	96.00	4.80	4.80	78.53
33	3	2	8	8	2	7	0.88	52.00	95.00	3.90	3.41	81.94
58	12	6	10	6	6	6	0.60	58.00	92.00	3.70	2.22	84.16
31	6	3	13	15	3	13	1.00	73.00	90.50	2.90	2.90	87.06
52	2	0	7	7	0	6	0.86	80.00	90.50	2.60	2.23	89.29
35	1	1	5	6	1	5	1.00	86.00	90.00	1.40	1.40	90.69
59	1	1	2	2	1	2	1.00	88.00	89.50	0.90	0.90	91.59
39	5	2	3	3	1	3	1.00	91.00	88.50	0.80	0.80	92.39
51	2	0	1	3	0	1	1.00	94.00	88.50	0.20	0.20	92.59
56	2	2	3	3	1	3	1.00	97.00	87.50	0.20	0.20	92.79
Negative/Not detectable/No consensus	138	156	2	3	138	2	—	100.00	9.50	—	—	—
Invalid	17	19	0	0	19	0	—	100.00	0.00	—	—	—

a Calculated as: cases remained positive after thresholds/cases ‐ reference.

b Based on HPV distribution among cases and controls after thresholds were applied.

A few very well‐characterized HPV assays are considered as comparator assays in validation studies, including Cobas 4800 and BD Onclarity (referred to as BD/Cobas) [30]. Table 4 compares the consensus HPV distribution in cases and controls with the HPV‐positives for the same types from BD/Cobas. The comparator assay testing using manufacturer‐defined thresholds achieved high ICC‐weighted sensitivity (96.61%) but much lower specificity (82.50%).

**Table 4:** HPV type distribution determined by BD/Cobas with consensus HPV distribution from Table 1 as Reference. Consensus on distribution of HPV types were recategorized to adapt the output of BD Onclarity assay. In case of positivity for multiple HPV types, the most oncogenic HPV type is listed following HPV16 >18 >45 > (33/58) >31 >52 > (35/39/68) > (56/59/66) >51 >Other HPV. ICC‐weighted sensitivity was calculated using PAF adapted from IARC [25].

HPV type	Control ‐ consensus	Controls – BD/Cobas	Case ‐consensus	Case‐ BD/Cobas	Controls ‐ BD/Cobas positives when consensus positive	Cases BD/Cobas positives when consensus positive	Sensitivity^b^	Cumulative sensitivity%^c^	Cumulative Specificity%^c^	PAF ICC%	Sensitivity with threshold%	Cumulative Sensitivity ICC weighted%
16	6	10	34	34	6	34	1.00	34.00	95.00	62.40	62.40	62.40
18	2	2	7	6	2	6	0.86	40.00	94.00	15.30	13.11	75.51
45	2	1	4	4	1	4	1.00	44.00	93.50	4.80	4.80	80.31
33/58	10	8	15	14	8	14	0.93	58.00	89.50	7.60	7.09	87.41
31	5	4	15	15	4	15	1.00	73.00	87.50	2.90	2.90	90.31
52	2	1	8	8	1	8	1.00	81.00	87.00	2.60	2.60	92.91
35/39/68	7	4	9	11	4	9	1.00	92.00	85.00	2.40	2.40	95.31
56/59/66	6	5	6	6	5	6	1.00	98.00	82.50	1.10	1.10	96.41
51	1	0	1	1	0	1	1.00	99.00	82.50	0.20	0.20	96.61
Other HPV^a^	11	—	0	—	—	—	—	99.00	82.50	—	—	—
Negative/Not detectable/No consensus	148	165	1	1	148	1	1.00	100.00	0.00			

a HPV 6, 40, 42, 44, 61, (44 & 61), 54, 61, 82.

b Calculated as: cases remained positive when consensus positive/cases—consensus.

c Calculated based on BD/Cobas output on HPV distribution among cases.

Table 5 compares the methods used by the different countries to the comparator (Table 4). In general, specificity of the different assays that used the proposed analytical detection thresholds were greatly improved compared to the comparator assay, whereas the ICC‐weighted sensitivity was only marginally less or the same (Table 5).

**Table 5:** Comparison of the performance results received using different HPV testing methods used by the different countries to the BD/Cobas and consensus results.

	Cumulative Sensitivity%	Cumulative Specificity%	ICC Weighted Cumulative Sensitivity%	Relative Cumulative Sensitivity%	Relative Cumulative Specificity%	Relative ICC Weighted Cumulative Sensitivity%
BD/Cobas^a^	99.00	82.50	96.61	ref	ref	ref
Consensus^b^	89.00	92.00	90.08	89.90	111.52	93.25
Belgium	88.00	91.00	92.55	88.89	110.30	95.79
France	83.00	93.50	87.60	83.84	113.33	90.67
Germany	92.00	89.50	93.36	92.93	108.48	96.64
Italy	91.00	92.00	82.91	91.92	111.52	85.82
Scotland	83.00	92.50	83.30	83.84	112.12	86.22
Slovenia	81.00	94.00	86.16	81.82	113.94	89.19
Sweden	93.00	90.00	94.58	93.94	109.09	97.90

a Results from Table 4.

b Results from Table 2.

## 4 Discussion

We explored optimal analytical detection thresholds of individual HPV types in HPV tests intended for cervical screening. Because even reference laboratory testing can show considerable variability, an international collaborative study with 10 participating countries was conducted using the same samples. The identified type‐specific analytical detection thresholds for the 12 oncogenic HPV types 16/18/31/33/35/39/45/51/52/56/58/59 could be used to greatly improve specificity (from 81.50% to 92.00%) with marginal loss of sensitivity (confer Tables 2 and 5). When analysis was restricted to the eight major oncogenic HPV types (16/18/31/33/35/45/52/58), specificity increased further to 94.50%. Given that HPV‐based screening is now a globally recommended public health policy, these improvements in specificity would correspond to > 10% of the global screening‐eligible population of women no longer being classified as screen‐positive, while potentially missing only a small proportion of the CIN2+ lesions with potential to progress to ICC.

A strength of this study is its international robustness, combining results from 10 countries/laboratories using several different assay platforms. Additionally, study samples were selected from a population‐based screening program, ensuring robust contextual data, while both semi‐quantitative and quantitative outputs enabled comprehensive analysis. Furthermore, expressing results in IU, traceable to WHO IS, ensures global comparability, both with current and future testing systems. However, several limitations should be noted. The study sample size was limited, resulting in a small number of observations for uncommon HPV types. Also, different screening programs may use different sample‐taking systems. The LBC samples used in the study (collected with cervical brush suspended in 20 ml ThinPrep medium) represent the most common sampling method today, but alternative sampling media and collection systems exist. Analytical thresholds should be able to accommodate also future changes in how the samples are collected. Most screening tests do not relate the HPV test results to the amounts of cells in the sample, but in order for the thresholds to be transferable across sampling systems, we also provide data on how they perform if the amount of HPV is expressed in relation to the number of human cells in the sample.

The WHO target product profile (TPP) outlines principles for HPV tests for screening that are appropriate for public health needs [31]. The TPP specifies high‐performance HPV tests to detect, as a minimum, types 16/18/31/33/35/45/52/58 and specifically recommend against including HPV types outside of the 12 HPV types formally established as oncogenic (such as HPV68 and 66). For absolute clinical sensitivity of CIN2+, a minimum standard of 90% was proposed. TPP also highlighted that, although current HPV tests generally achieve very high sensitivity, specificity remains suboptimal. Our findings align with the TPP as both reference laboratory testing without thresholds and comparator assay testing had very high sensitivity, but suboptimal specificity. Including HPV types that were not formally classified as oncogenic greatly reduced specificity (to 74.00%), with negligible gains in sensitivity.

Older validation methods are dependent on clinical samples with longitudinal follow‐up for disease, making validation slow and expensive. Furthermore, the most oncogenic HPV types are becoming less common in CIN2+ in vaccinated birth cohorts. Present‐day series of CIN2+ will contain non‐vaccine HPV types of limited relevance for cervical cancer. Indeed, we found a decline of HPV16 in CIN2+ among birth cohorts that had been offered vaccination, necessitating weighting of the sensitivity according to the importance of the different HPV types for invasive cervical cancer, as assessed in the era before widespread HPV vaccination.

Reference laboratory testing from all participating countries had huge improvements in specificity compared to comparator assay testing, but only three lab achieved the TPP criterion of both absolute and relative (compared to comparator assay testing) sensitivity of > 90%. However, regular issuing of proficiency panels has found quite large improvements in sensitivity over time [15, 16, 17, 18, 19, 20, 21, 22, 23], implying that it will not be difficult to both improve specificity and achieve the WHO TPP thresholds for sensitivity.

Many HPV tests define a threshold for HPV positivity using Ct values from a PCR reaction. However, different PCR‐based tests amplify at different efficiencies and Ct values from one assay cannot be translated to Ct values for another. Also, Ct values are not linearly related to the amount of virus. Calculating the amount of virus using a standard curve of samples with defined amounts of virus in IU reduces these problems and increases international comparability. Since nearly all assays used in this study detected HPV types separately, threshold values for detection could be applied to a single HPV type.

This study included only clinician‐taken samples. Therefore assessment of optimal thresholds for detection in self‐collected samples would require an entirely new study as the converted Ct outputs for different virus concentrations may differ from those of samples taken by healthcare personnel. Furthermore, pre‐analytical variations in laboratory workflow, such as the extraction method, the suspension volume of the analysis platforms, and operator issues with sample collection may also affect the virus amount in samples. To minimize accuracy and precision errors, standards should be analyzed multiple times to determine median Ct values and estimate virus quantities in samples in relation to standard curves. As the total amount of cervical cells obtained may vary enormously, most HPV DNA screening tests do not express the amount of virus present in relation to the number of cervical cells obtained. Performance is somewhat better if simply relating the amount of virus to the input volume of the test, but the alternative to relate the thresholds to the number of human cells present has advantages in terms of theoretically being more transferable to different settings.

The current study is a first step towards defining virological endpoints for evaluation of HPV screening tests. The limited sample size was a necessity given the large number of participating laboratories. In further exploration of virological endpoints, many different settings are likely to be able to investigate large laboratory databases from real‐life screening programs which would provide data on the robustness of the required analytical threshold of detection for HPV type specific virus amounts proposed here.

In conclusion, this study defined analytical detection thresholds for HPV genotypes that achieved much higher specificity while maintaining adequate sensitivity in HPV‐based cervical screening. Definition of which amount of virus, traceable to IS, that should be detectable for optimal sensitivity and specificity in cervical screening could facilitate the development, validation and quality assurance of HPV testing methods.

## Ethics Statement

Ethical approval was granted by the national ethical review agency of Sweden (DNR2023‐02457‐01, DNR2025‐08540‐02). The ethical review agency determined that informed consent was not required.

## Conflicts of Interest

KC's, LC's, CB's, and AC's institution has received research funding or gratis consumables to support research from the following commercial entities in the last 3 years: Abbott, Euroimmun, GeneFirst, Qiagen, Hiantis, Seegene, Roche, Hologic, Barinthus Biotherapeutics PLC & Daye. KC has attended advisory board meetings for Hologic, Abbott Becton Dickinson and Barinthus Biotherapeutics PLC (no personal renumeration received; UK travel supported for Hologic and Abbott). KC has received support from COPAN to attend a scientific conference.

AO has received reimbursement of travel expenses for attending conferences and honoraria for speaking from Abbott Molecular, Qiagen, and Seegene. MP declares no personal conflicts of interest. MP's and AO's institution received research funding, free‐of‐charge reagents, and consumables to support research in the last three years from Qiagen, Seegene, Abbott, and Roche, all paid to their employer (no personal remuneration received).

Other authors report no conflicts of interest.

## Declaration of Generative AI and AI‐Assisted Technologies in the Manuscript Preparation Process

During the preparation of this work the first author, EY AI‐assisted tools (Microsoft Copilot and ChatGPT, OpenAI) in order to assist development and debugging of analysis codes in the script. However, the tools were not used to generate or interpret data, or on any scientific decisions. The code and output of the analysis were reviewed and verified by EY.

## Supporting information

Supporting File

## Data Availability

The individual level anonymized data will be freely made available at B2Share (b2share.eudat.eu).
