# Supplementary material for: International Collaborative Study on Human Papillomavirus Analytical Thresholds for Sensitivity and Specificity in Cervical Screening
Source: J Med Virol. 2026 Apr 25;98(5):e70936. doi: 10.1002/jmv.70936 (PMC13109973; doi:10.1002/jmv.70936)
Supplement: Supplementary file 1 — Supporting File [file JMV-98-e70936-s001.docx]

**SUPPLEMENTARY MATERIAL**

*HPV Testing Methods*

NRL Sweden used Cobas 4800 (Roche Diagnostics, Basel, Switzerland) or BD Onclarity™ HPV Assay (Becton, Dickinson and Company, Eysins, Switzerland) for the cervical screening program. The reference method for HPV quantification used the Hamilton® Microlab Star (Hamilton Bonaduz AG, Bonaduz, Switzerland) automated DNA extraction method with the Mag-Bind® Universal Pathogen 4x96 (Omega Bio-Tek, Leverkusen, Germany) kit and HPV analysis on the Quant Studio™ 3 Real-Time PCR system (Thermo Fisher Scientific Applied Biosystems™, Darmstadt, Germany), targeting the E6/E7 region of HPV16, 18, 31, 33, 35, 39, 45, 51, 52, 56, 58, 59, respectively. Real-time PCR reactions in 20 µL volumes contained 50-100 ng of genomic DNA, GoTaq® Probe Master Mix (Promega Corporation, Mannheim, Germany), 0.5 µM of each forward and reverse primer, and 0.25 µM of HPV QSY-probe. Each run included DNA-free water (non-template control). Initial denaturation at 95°C for 2 minutes was followed by 45 cycles of 95°C for 3 seconds and 60°C for 30 seconds. Cut offs for detection and baseline were chosen by the Quant Studio™ 3 software. Standards were run as triplicates while study samples were run as duplicates and the mean amounts of HPV recorded.

The NRL of Australia used Roche MagNA Pure 96 DNA (Roche Diagnostics, Basel, Switzerland) with Viral NA Small Volume Kit (Roche Diagnostics, Basel, Switzerland) for DNA extraction and performed HPV detection using Anyplex™ II HPV HR Detection (Seegene Inc, Seoul, South Korea) and quantitation on an in-house droplet digital PCR targeting the E6 gene on the Bio-Rad QX100/QX200 system (Bio-Rad Laboratories, California, USA) (manuscript in preparation); NRL of Belgium used Chemagic 360 system (Revvity, Turku, Finland) for automated DNA extraction from liquid-based cytology samples, employing magnetic bead-based technology and for HPV analysis the in-house developed RIATOL quantitative real-time PCR genotyping assay (AML Laboratory, Antwerp, Belgium) that individually targets 18 HPV genotypes (HPV6, 11, 16, 18, 31, 33, 35, 39, 45, 51, 52, 53, 56, 58, 59, 66, 67, and 68) using eight multiplex reactions per sample was performed at ultra-low reaction volumes on the LightCycler instrument (Roche Diagnostics, Basel, Switzerland); NRL of Germany used Roche Magna Pure DNA/Viral NA LV 2.0 (Roche Diagnostics, Basel, Switzerland) for extracting DNA from the samples and performed HPV analysis using Allplex™ HPV28 Detection platform (Seegene Inc, Seoul, South Korea) as well as HPV type-specific in-house real time PCR; NRL of France used Microlab Nimbus (Hamilton Bonaduz AG, Bonaduz, Switzerland) with the NucleoMag Dx Pathogen kit (Macherey Nagel, Düren, Germany) for DNA extraction and in-house real-time PCR assay for HPV analysis; NRL Italy used Microlab Nimbus for DNA extraction and OncoPredict HPV QT assay (Hiantis, Milan, Italy) for HPV analysis; NRL of Scotland performed DNA extraction using Universal extraction system with STARMag Universal Cartridge kit (Seegene Inc, Seoul, South Korea) and conducted HPV analysis completed HPV analysis using Allplex™ HPV28 Detection platform; NRL of Slovenia used QIAamp DNA Mini Kit (Qiagen, Hilden, Germany) for DNA extraction and Allplex™ HPV HR for HPV testing; NRL USA analyzed the samples for HPV using Typeseq2 (Frederick National Laboratory for Cancer Research, Maryland, United States); participating laboratory from Türkiye used in-house DNA isolation kit (DNA Elixir Smear, Izmir, Türkiye) to extract DNA from the samples and in-house real-time PCR assay (CarciScan HPV PCR Assay, Izmir, Türkiye) for HPV analysis.

**Supplementary Table I:** Assay and input volume information used for quantification of HPV type specific virus amounts by laboratories.

| Countries/Laboratories | Extraction Method | Input volume for extraction (µl) | Extracted Volume (µl) | HPV analysis method | Input volume of DNA eluate for HPV analysis (µl) |
| --- | --- | --- | --- | --- | --- |
| Belgium | Chemagic 360 system | 1000 | 100 | In-house RIATOL quantitative real-time PCR on the LightCycler instrument | 2 |
| Germany | Magna Pure DNA/Viral NA LV 2.0 | 500 | 100 | Allplex™ HPV28 HPV positive samples subjected to in-house real time PCR for quantification | 5* |
| France | Microlab Nimbus with the NucleoMag Dx Pathogen kit | 200 | 100 | In-house real-time PCR | 5 |
| Italy | Microlab Nimbus | 200 | 100 | OncoPredict HPV QT assay | 5 |
| Scotland | Universal extraction system with STARMag Universal Cartridge kit | 300 | 100 | Allplex™ HPV28 Detection | 10 |
| Slovenia | QIAamp DNA Mini Kit | 200 | 50 | Allplex™ HPV HR | 5 |
| Sweden | Hamilton® Microlab Star with the Mag-Bind® Universal Pathogen 4x96 kit | 250 | 200 | BD/Cobas HPV positive samples subjected to Quant Studio™ 3 Real-Time PCR system for quantification | 9 |

*Input volume of 2 µl and 5 µl was used for the analysis of serially diluted standards and study samples, respectively.
